# Curcumin inhibits APOE4-induced injury by activating peroxisome proliferator-activated receptor-γ (PPARγ) in SH-SY5Y cells

**DOI:** 10.22038/ijbms.2020.47184.10858

**Published:** 2020-12

**Authors:** Minghui Wang, Jiejian Kou, Chunli Wang, Xiuying Yu, Xinmei Xie, Xiaobin Pang

**Affiliations:** 1 Pharmaceutical Institute, Pharmaceutical College of Henan University, Kaifeng 475004, China; 2 Agricultural College of Inner Mongolia University for Nationalities, Tongliao, 028043, China

**Keywords:** Apolipoprotein E4, Curcumin, Neuroinflammation, NF-KappaB, PPAR gamma

## Abstract

**Objective(s):**

The human apolipoprotein E4 (APOE4) is associated with various brain injuries and neurodegenerative changes. Curcumin is an active ingredient isolated from the root of turmeric and is believed to have therapeutic effects on neurodegenerative diseases. The aim of this study was to investigate the effects of curcumin on APOE4-induced neurological damage and explore its molecular mechanisms.

**Materials and Methods:**

SH-SY5Y cells were pretreated with curcumin for 24 hr and transfected with human APOE4 gene using Lipofectamine 2000. Then, the effect of curcumin on the transfected cells was detected by ELISA, immunofluorescence staining and Western blot.

**Results:**

The production or expression of proinflammatory cytokines and proteins, including tumor necrosis factor-α (TNF-α), interleukin-1β (IL-1β), nitric oxide (NO), inducible nitric oxide synthase (iNOS) and cyclooxygenase-2 (COX-2) was significantly increased in SH-SY5Y cells transfected with APOE4, and curcumin inhibited APOE4-induced cellular inflammatory damage. Western blot analysis showed that, after transfection with APOE4, the expression of total nuclear factor kappa B (NF-κB) p65 and p-NF-κB p65 in the nucleus was increased, and curcumin inhibited the nuclear translocation of p65. The overexpression of APOE4 inhibited the expression of peroxisome proliferator-activated receptor-γ (PPARγ), whereas curcumin reversed and increased the expression of PPARγ protein. Down-regulating PPAR-γ with the inhibitor GW9662 and the shPPARγ gene confirmed that the NF-κB signaling pathway was inhibited by PPARγ.

**Conclusion:**

This study suggests that APOE4 overexpression can induce cellular inflammatory damage, and pretreatment of curcumin could exert an anti-inflammatory effect by upregulating the expression of PPARγ to inhibit the activation of NF-κB signaling pathway.

## Introduction

With the rapid increase of aging in human society, the incidence of neurodegenerative diseases, such as Alzheimer’s disease (AD) and Parkinson’s disease (PD), is increasing year by year (1). These diseases are characterized by the gradual loss of neuronal structure or function and even death (2). At present, neurodegenerative diseases are incurable and their etiologies are still unclear, which bring a serious threat to human health and daily life.

Neuro-inflammation occurs widely in a variety of neurological diseases, such as AD, depression, cognitive impairment after craniocerebral injury, etc. (3). In the past few decades, it has been recognized that abnormal inflammatory processes in the central nervous system can lead to neurological dysfunction (4). Therefore, inhibiting the abnormal occurrence of neuro-inflammation has a guiding role in the treatment of diseases.

Clinical and preclinical studies have shown that the human apolipoprotein E4 (APOE4) is associated with poor outcomes of a variety of brain injuries and neurodegenerative changes (5). Especially, APOE4 has long been considered as an important risk factor for AD. Previous studies have suggested that APOE4 promotes the aggregation of Aβ and accelerates the formation of senile plaques (6); some researchers have found that APOE4 can aggravate the neurological damage caused by the tau protein (7). In recent years, it has been noted that APOE4 is closely related to neuro-inflammation. The expression of the proinflammatory factors, such as tumor necrosis factor-α (TNF-α) and interleukin-1β (IL-1β), in systemic and brain tissues were significantly increased in APOE4-targeted replacement mice compared to APOE3-targeted replacement mice after the intravenous administration of lipopolysaccharides (LPS) (8). In BMT-APP/PS1 mice, E4 is more strongly associated than E3 with impaired spatial memory, and the expression of proinflammatory factors is higher (9). Another study showed that human APOE4 increases microglia reactivity with Aβ plaques in APOE4-targeted replacement mice (10). Therefore, the overexpression of APOE4 may promote the release of inflammatory factors and trigger an inflammatory response leading to AD.

Peroxisome proliferator-activated receptor γ (PPARγ) belongs to the nuclear hormone receptor family and is a key transcription factor that regulates inflammation. PPARγ can effectively inhibit the activity of nitric oxide synthase (NOS) and the production of reactive oxygen species (11). The activation of PPARγ may be closely related to the inhibition of the nuclear factor kappa B (NF-κB) signaling pathway. Studies have shown that chrysophanol could inhibit the LPS-induced inflammatory response by activating PPARγ/NF-κB pathway in RAW264.7 cells (12). In the brains of AD patients, the expression of PPARγ is significantly decreased, but the specific mechanism is still unclear. 

**Figure 1:**
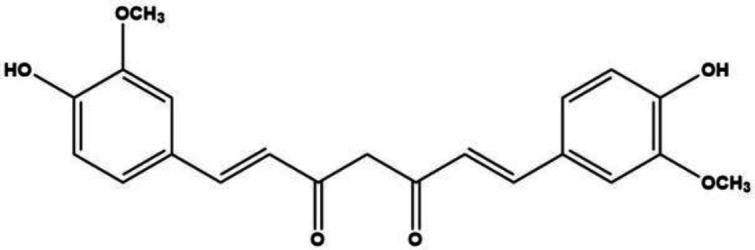
Chemical structures of curcumin

Curcumin is a special diketone compound extracted from the roots of turmeric (13). The chemical structure is shown in Figure 1. It has been used as a condiment for food for centuries, especially in Southeast Asia (14). According to the epidemiological analysis of the original Indian population, curcumin has a strong potential for the treatment of AD. Statistical analysis has shown that the incidence of AD in individuals that use curcumin for long-term is 4.4 times lower than that in the United States (15). According to toxicity studies, it is quite safe even at high doses (up to 12 g every day in humans) (16). The pharmacological effects of curcumin are mediated by transcription factors, enzymes, growth factors, neurotransmitter receptors, growth factor receptors, cytokine receptors, inflammatory mediators and a large number of protein kinases with antioxidant (17, 18), anti-inflammatory, anticancer, and anticardiovascular activities (19, 20). In addition, curcumin is a strong antioxidant compound that has a strong free radical scavenging effect. However, whether curcumin plays a role in AD by inhibiting neuro-inflammation remains unclear. Therefore, in this study we investigated the protective effect of curcumin on the APOE4-mediated injury in SH-SY5Y cells and explored the role that PPARγ plays in this process. The results of this study may also provide new ideas and strategies for studying the pathogenesis of AD and developing drugs for this type disease. 

## Materials and Methods

***Plasmid extraction***

Transforming bacteria containing GV230-CON (GV-Con), GV230-APOE4 (GV-APOE4), CON-RNAi (Con shRNA), and PPARγ-RNAi (PPARγ shRNA) (all from Jikai Gene, Shanghai, China) were inoculated in LB medium containing kanamycin or ampicillin and rotated for 14-16 hr at 230 rpm at 37°C. The plasmid was extracted in 5 ml of bacterial solution and centrifuged at room temperature. The plasmid was extracted according to the instructions of the endotoxin-free plasmid kit (Kangwei Century Biotechnology, Shanghai, China), and the plasmid concentration was detected by an ultramicrospectrophotometer after successful extraction (21).

***Cell culture***

Neuroblastoma SH-SY5Y cells (Cell Bank of the Chinese Academy of Sciences, Shanghai, China) were cultured in Dulbecco’s modiﬁed Eagle’s medium (DMEM) containing 8% fetal bovine serum (Sigma, St. Louis, USA) in a 37 °C and 5% CO_2_ incubator. On the day before transfection, SH-SY5Y cells growing in the logarithmic phase were seeded in 6- or 96-well plates, and curcumin (Sigma, USA), which was dissolved in DMSO at a final concentration less than 0.1%, was added to the wells for 24 hr. 

***Cell transfection***

Transfection was performed according to the manufacturer’s instructions using Lipofectamine 2000 (Invitrogen, USA) when the cells reached 80% conﬂuence. The cells were cultured in an incubator at 37 °C and 5% CO_2_ for 48 hr and observed by fluorescence microscopy (Olympus, Japen).

***Cell viability assay***

Cell viability was measured by the CCK-8 method. The cells were digested with trypsinase and seeded into 96-well plates. After pretreatment with curcumin and transfection, CCK-8 (Dojindo Laboratories, Kumamoto, Japan) was added to each well. The cells were incubated in the incubator for 3-4 hr, and the OD value of each well was measured using a multifunction microplate reader (Manufacturer Perkin Elmer Singapore Pte Ltd., Singapore).

***Cell proliferation measure***

The cells were digested with trypsin and inoculated into 96-well plates. Curcumin and plasmid were added to the cells. After 48 hr, the proliferation ability of the cells was tested according to the instructions of the 5-ethynyl-2’-deoxyuridine (EDU) kit (RIBBIO, Guangzhou, China) (22, 23). 

***Inflammatory mediator measurements***

Cells were seeded into 6-well plates, and curcumin and plasmid were added for 48 hr. The supernatant was collected, and the levels of TNF-α and IL-1β were determined by an ELISA kit (Jiancheng Bioengineering Institute, Nanjing, China) according to the manufacturer’s instructions. The release concentration of nitric oxide (NO) was detected by the kit (Jiancheng Bioengineering Institute, Nanjing, China). The calibration curve of the sodium nitrite standard was used to monitor NO production.

***Immunofluorescence***

Cells were inoculated into 6-well plates, curcumin and plasmid were added for 24 hr, and the cells were cultured in a confocal chamber (Nest, Jiangshu, China). After 24 hr, the cells were fixed in a 4% paraformaldehyde solution for 10 min. Then, the cells were permeabilized with 0.1% Triton X-100 for 5 min. After treatment with a phosphate-buffered saline (PBS) solution containing 5% bovine serum albumin (BSA) for 1 hr, the cells were incubated overnight with an anti-NF-κB p65 antibody (1:400, Cell Signaling Technologies, Danvers, MA, USA) at 4 °C and then incubated with an Alexa Fluors 594-conjugated fluorescent secondary antibody (1:500, Proteintech Group, Inc., Wuhan, China) at room temperature for 1 hr. The nuclei were stained with DAPI (10 µg/ml) for 10 min. After washing with PBS, the samples were analyzed by laser confocal microscopy (NIKON A1-N-SIM, Japan) (24).

***Western blot analysis***

Protein concentrations were measured using a protein assay kit according to the manufacturer’s instructions. The extracted proteins were separated by SDS-polyacrylamide gel electrophoresis and then transferred to a membrane for 30 min. The membrane was blocked with 5% skim milk and then incubated at 4 °C overnight with the following primary antibodies: anti-iNOS, anti-COX-2, anti-APOE4, anti-p65, anti-p-p65, and anti-PPARγ (all purchased from Cell Signaling, Boston, MA, USA, 1:1000). Then, the membrane was washed with TBST (Tris-buffered saline and Tween 20) three times and incubated with a second antibody (sheep anti-mouse or sheep anti-rabbit, 1:2000) for 2 hr. The ECL method was used to develop the film, and dark room exposure analysis and ImageJ 2x analysis software (National Institutes of Health, Bethesda, MD) were used to analyze the gray values (25).

***Statistical analysis***

All experiments were performed at least three times. The data were analyzed by SPSS 17.0 software. The experimental data are expressed as the mean ± SD, and the T values of the independent samples were used to determine the *P* values. *P*<0.05 was considered statistically signiﬁcant.

## Results

***The human APOE4 gene was overexpressed in SH-SY5Y cells as a result of liposome transfection***

**Figure 2:**
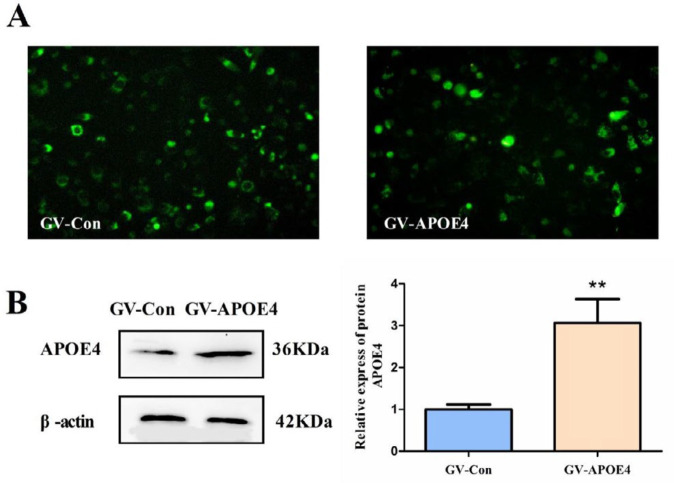
The Human APOE4 gene was overexpressed in SH-SY5Y cells as a result of liposome transfection. After 48 hr of transfection, APOE4 protein expression was detected by immunofluorescence and western blot. (A) Representative images of the expression of the APOE4 gene, as detected by immunofluorescence (100×). (B) The expression of the APOE4 protein in transfected SH-SY5Y cells was detected by western blot. The data represent the means±SD from three independent experiments. Compared to GV-Con, **P<0.01

The human APOE4 gene was transfected into SH-SY5Y cells with Lipofectamine 2000. After transfection with GV230-APOE4 for 48 hr, the expression of APOE4 was examined by immunofluorescence and western blot analysis. Because the plasmid vector contains green fluorescent protein (GFP), it can be observed by fluorescence microscopy. As shown in Figure 2A, the GV230-CON and GV230-APOE4 groups showed strong fluorescence, suggesting that the plasmid transfection was successful. The western blot results showed that the level of APOE4 protein in the SH-SY5Y cells transfected with GV230-APOE4 was significantly higher than that in the GV230-CON group. The above results suggest that the exogenous APOE4 gene was overexpressed in SH-SY5Y cells.

***Effect of curcumin on*** ***the viability*** ***of SH-SY5Y cells overexpressing APOE4***

**Figure 3:**
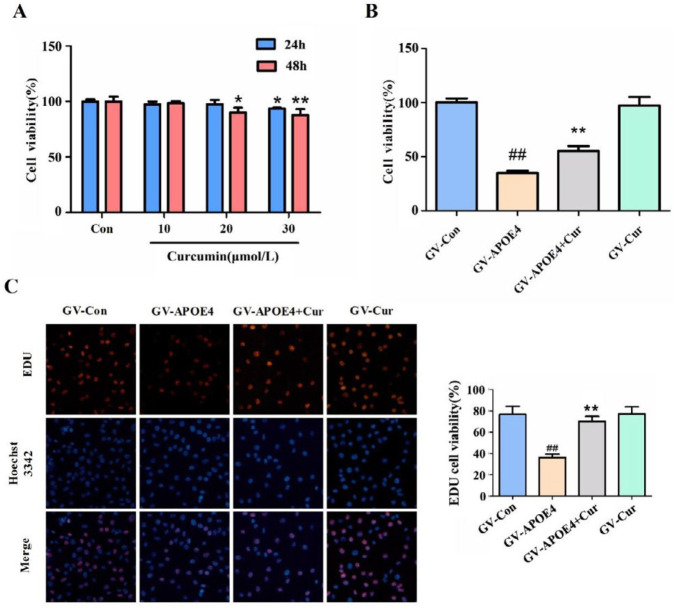
The effect of curcumin on the viability of SH-SY5Y cells overexpressing APOE4. Forty-eight hour after transfection, the effects of different treatments on cell survival and proliferation were detected by the CCK-8 and EDU kits, respectively. (A) The determination of the concentration and treatment time of curcumin. (B) Curcumin reversed the reduced survival rate induced by APOE4 overexpression in SH-SY5Y cells. (C) Curcumin inhibited the decrease in SH-SY5Y proliferation induced by APOE4 transfection. The data represent the means±SD from three independent experiments. Compared to the GV-Con group, ##*P*<0.01; compared to GV-APOE4, **P<0.01

The CCK-8 method was applied to determine the appropriate concentration (0~30 μmol/l) and pretreatment time (24 hr, 48 hr) of curcumin in SH-SY5Y cells. According to the experimental results (Figure 3A), 10 μmol/l curcumin for 24 hr was used for subsequent experiments. After curcumin treatment and plasmid transfection, the CCK-8 assay was used to detect cell viability. Compared with that of the GV-con group, the survival rate of the GV-APOE4 group was significantly decreased. After curcumin treatment, the survival rate increased significantly (*P*<0.01, Figure 3B).

To further verify the protective effect of curcumin on APOE4-induced cells, an EDU kit was used to detect cell proliferation. As shown in Figure 3C, 48 hr after APOE4 gene transfection, SH-SY5Y cell proliferation was significantly inhibited (*P*<0.01). However, pretreatment of curcumin significantly promoted cell proliferation (*P*<0.01).

***Curcumin inhibited the release of inflammatory factors in SH-SY5Y cells*** ***transfected*** ***with APOE4***

**Figure 4:**
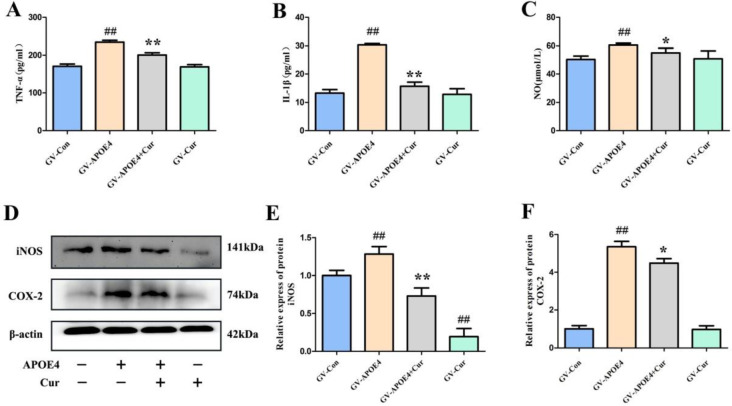
Effect of curcumin on the release of inflammatory factors in APOE4-induced SH-SY5Y cells. The secreted levels of TNF-α (A), IL-1β (B) and NO (C) were detected by ELISA and NO kit. (D) The protein expression levels of iNOS and COX-2 were detected by western blot assay. (E-F) Quantification of western blot data. Protein expression was normalized to expression of β-actin, and relative densities were normalized against the GV-Con group. The data represent the mean±SD from 3 independent experiments. Compared to GV-Con, ##*P*<0.01; compared to GV-APOE4, *P<0.05, **P<0.01

To investigate the relationship between APOE4 and neuro-inflammation and the role of curcumin, we examined the release of inflammatory factors. As shown in Figure 4, after transfection with APOE4, the levels of TNF-α, IL-1β and NO and the protein expression of inducible NOS (iNOS) and COX-2 in SH-SY5Y cells were significantly increased (*P*<0.01). Curcumin significantly inhibited the increase in these proinflammatory factors.

***Curcumin inhibited the*** ***APOE4-induced*** ***activation of the NF-κB signaling pathway***

**Figure 5:**
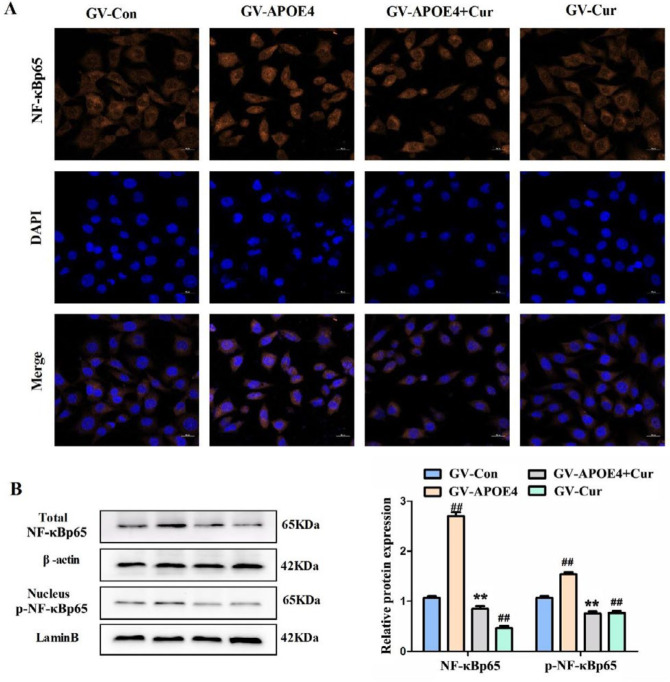
Effect of curcumin on the APOE4-induced NF-κB pathway in SH-SY5Y cells. (A) Curcumin inhibited the APOE4 overexpression-induced transport of NF-κB p65 from the cytoplasm to the nucleus, as detected by laser confocal microscopy (scale bar=50 μm). (B) Total NF-κB p65 and nuclear p-p65 protein expression in APOE4-transfected cells, as detected by western blot. (C) The quantification of the western blot data. Protein expression was normalized to that of β-actin or lamin B, and the relative densities were normalized to those of the GV-con group. The data represent the mean±SD from 3 independent experiments. Compared to GV-Con, ##*P*<0.01; compared to GV-APOE4, **P<0.01. NF-κB: Nuclear factor kappa B

The NF-κB signaling pathway plays an important role in neuro-inflammation. To investigate the effect of curcumin on the NF-κB pathway induced by APOE4, we examined the nuclear translocation of NF-κB p65 and its protein expression in SH-SY5Y cells transfected with APOE4. As shown in Figure 5A, Alexa Flour 594 staining (orange) indicates the location of the NF-κB p65 antibody, DAPI staining indicates the location of the nucleus (blue), and the combined images show the nuclear translocation of the NF-κB p65 protein. As expected, APOE4 transfection promoted the transfer of p65 into the nucleus, while curcumin inhibited the nuclear translocation of NF-κB p65. Western blot assays validated the fluorescence results. The expression of total p65 and nuclear p-p65 in APOE4-transfected cells was significantly increased, while curcumin inhibited the above changes. These results indicate that the overexpression of APOE4 activates the NF-κB signaling pathway in cells, whereas curcumin inhibits the APOE4-induced activation of this pathway.

***Effect of curcumin on PPARγ expression in SH-SY5Y cells overexpressing APOE4***

The activation of PPARγ is associated with inflammation and oxidative stress in nerve cells. We detected the expression of PPARγ in APOE4-transfected cells. The results showed that the expression of the PPARγ protein was significantly decreased in the APOE4-transfected group, while curcumin reversed and upregulated the expression of PPARγ.

***Curcumin inhibited the activation of NF-kB signaling through the PPARγ pathway in APOE4-transfected cells***

To determine whether curcumin inhibits NF-κB signaling through PPARγ, GW9662, a specific inhibitor of PPARγ, was used. Consistent with the above results, the overexpression of APOE4 downregulated PPARγ expression and increased the accumulation of p-p65 in the nucleus, while curcumin inhibited the above changes; however, GW9662 reversed the abovementioned effects of curcumin in transfected cells (Figure 7). 

Next, shRNA technology was used to downregulate PPARγ expression to further investigate its impact on the NF-κB signaling pathway. As shown in Figure 8, shRNA silenced PPARγ expression and increased the expression of total NF-κB p65 and nuclear p-p65. 

These results suggest that curcumin upregulated the expression of PPARγ, thereby inhibiting the activation of NF-κB signaling.

## Discussion

In this study, we found that the overexpression of APOE4 significantly increased the levels and expression of inflammatory cytokines and protein, including TNF-α, IL-1β, NO, iNOS and COX-2, suggesting that APOE4 overexpression causes an inflammatory response in nerve cells. Curcumin effectively inhibited APOE4-induced inflammatory damage by upregulating the expression of PPARγ and inhibiting NF-κB signal pathway. 

APOE4 is a major genetic risk factor of a variety of inflammatory metabolic diseases, such as atherosclerosis, diabetes, and AD (26). As the strongest genetic risk factor for AD, APOE4 plays a role in neuro-inflammation, and this role has attracted increasing attention in recent years and has been used as a new target of drug therapy (27, 28). The overexpression of APOE4 exacerbates the accumulation of Aβ protein, activates astrocytes and microglial cells and produces a large number of inflammatory factors, such as IL-1, IL-6 and TNF-α, which ultimately leads to neuronal apoptosis or death (29-32). Microglial cells cultured *in vitro* from APOE4-targeted replacement mice are more active than those from APOE3-targeted replacement mice, and E4 can produce higher levels of proinflammatory cytokines, including TNF-α and IL-6 (33). In this study, APOE4 was overexpressed in SH-SY5Y cells by liposome transfection, and the levels of TNF-α, IL-1β, NO and the expression of iNOS and COX-2 were significantly increased, suggesting that the overexpression of APOE4 induces an inflammatory response in cells.

Curcumin is a special diketone compound extracted from the rhizomes of *Zingiberaceae*. *In vivo* and *in vitro* studies have shown that curcumin has potentially important biological effects, including anti-inflammatory and antioxidant effects and is considered as a potential candidate drug for the treatment of AD. After being fed with curcumin for 6 months, the brains of AD model (APPswe) mice exhibited decreased levels of Aβ protein and inflammatory factors (34). In human acute leukemia cells (HTP-1), curcumin reduces the production of the cytokines IL-6 and TNF-α induced by Aβ protein and inhibits the phosphorylation of mitogen-activated protein kinase (MAPK) and extracellular regulated protein kinase 1/2 (ERK1/2) (35). The results of this study showed that curcumin reduced the production of proinflammatory factors induced by the overexpression of APOE4, suggesting that curcumin can inhibit the inflammatory response induced by APOE4.

NF-κB is an important transcriptional regulator that regulates the expression of various inflammatory factors after they are activated. Therefore, the inhibition of NF-κB activation is considered to be an important target of anti-inflammatory therapy (36-39). A study showed that the pretreatment of microglia with 10 μM curcumin inhibits the release of proinflammatory cytokines by inhibiting the NF-κB and activator protein 1 (AP-1) signaling pathways (40). In addition, curcumin downregulates LPS-induced COX-2 overexpression in BEAS-2B cells by inhibiting the NF-κB signaling pathway (41). In this study, we found that the overexpression of APOE4 promoted an increase in the expression of total p65 and nuclear p-p65 protein, while curcumin treatment inhibited the activation of the NF-κB pathway. Our results indicate that APOE4 causes intracellular inflammation by activating the NF-κB signaling pathway, and the therapeutic effect of curcumin may occur through the regulation of NF-κB.

A number of studies have shown that PPARγ may be involved in the inflammatory response and indirectly inhibit NF-κB by activating transcription factors, thus playing a crucial role in inflammation (42, 43). PPARγ can reduce γ-interferon (IFN-γ)- and LPS-induced cytokine production by inhibiting signal transducer and activator of transcription-1 (STAT1), NF-κB and AP-1 (44). The results of this study showed that the PPARγ level was decreased in cells transfected with APOE4, and curcumin treatment increased the expression of PPARγ, suggesting that curcumin may play an anti-inflammatory role by increasing the level of PPARγ protein. Subsequently, we investigated the relationship between PPARγ and the NF-κB signaling pathway. The expression of PPARγ protein was inhibited by the PPARγ inhibitor GW9662. The results confirm that GW9662 reversed the inhibition of curcumin on the expression of p-p65 in APOE4-overexpressing cells, suggesting that PPARγ might play an anti-inflammatory role by inhibiting the NF-κB signaling pathway. Next, shRNA was used to silence the PPARγ protein and further verified that the NF-κB signaling pathway is regulated by PPARγ. 

APOE4: apolipoprotein E4

APOE4: apolipoprotein E4

TNF-α: Tumor necrosis factor-α, IL-1β: Interleukin-1β, NO: Nitric oxide, iNOS: Inducible nitric oxide synthase, COX-2: Cyclooxygenase-2; APOE4: apolipoprotein E4; TNF-α: tumor necrosis factor-α

**Figure 6:**
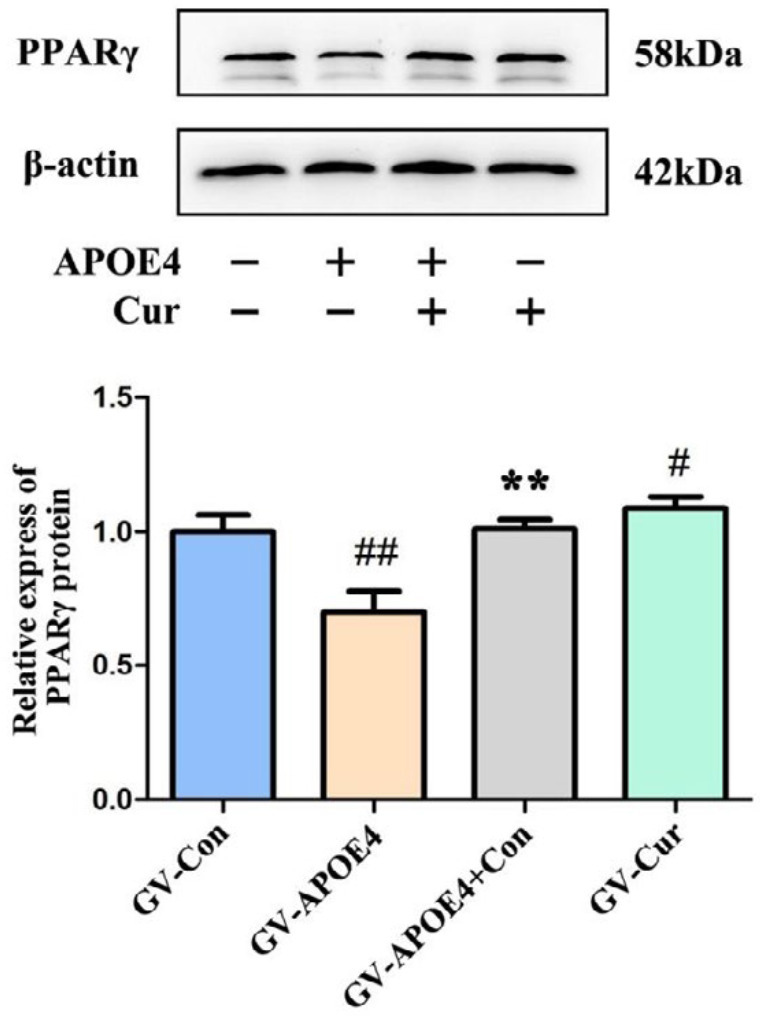
Effects of curcumin on the expression of PPARγ in SH-SY5Y cells transfected with APOE4, as detected by western blot. Protein expression was normalized to that of β-actin, and the relative densities were normalized to those of the GV-Con group. The data represent the mean±SD from three independent experiments. Compared to GV-Con, #*P*<0.05;##*P*<0.01; compared to GV-APOE4, **P<0.01

PPARγ: Peroxisome proliferator-activated receptor-γ; APOE4: apolipoprotein E4

**Figure 7:**
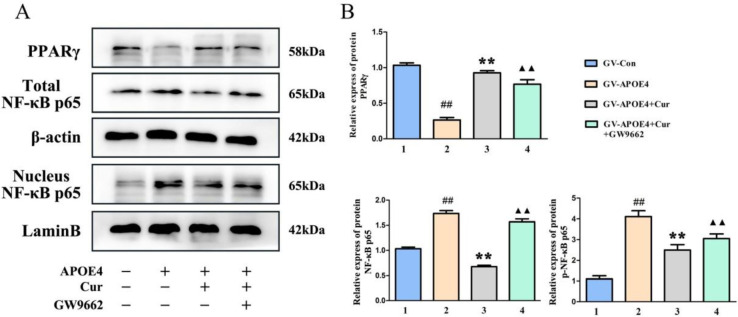
Effects of GW9662 on PPARγ and NF-κB p65 in APOE4-transfected cells. Pretreatment with curcumin and the inhibitor GW9662 for 24 hr and APOE4 gene transfection for 48 hr. (A) The expression of PPARγ and NF-κB p65 proteins was analyzed by western blotting. (B) The quantification of the western blot data. The data represent the mean±SD from 3 independent experiments. Compared to GV-Con, ##*P*<0.01; compared to GV-APOE4, **P<0.01; compared to GV-APOE4+Cur, *P*<0.01

PPARγ: Peroxisome proliferator-activated receptor-γ; NF-κB: Nuclear factor kappa B

**Figure 8:**
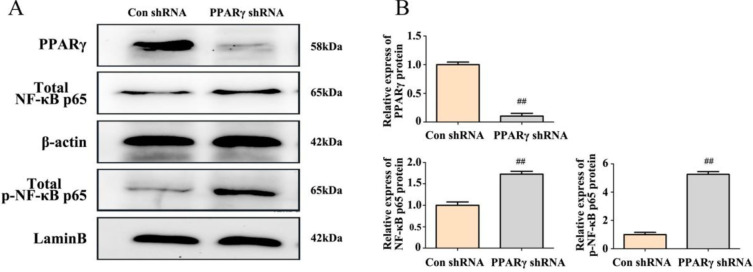
Effects of shPPARγ on NF-κB p65 in SH-SY5Y cells. After transfection with shPPARγ for 48 hr. (A) The expression of PPARγ and NF-κB p65 proteins was analyzed by western blot. (B) The quantification of the western blot data. The data represent the mean±SD from 3 independent experiments. Compared to Con, ##*P*<0.01

PPARγ: Peroxisome proliferator-activated receptor-γ; NF-κB: Nuclear factor kappa B

**Figure 9:**
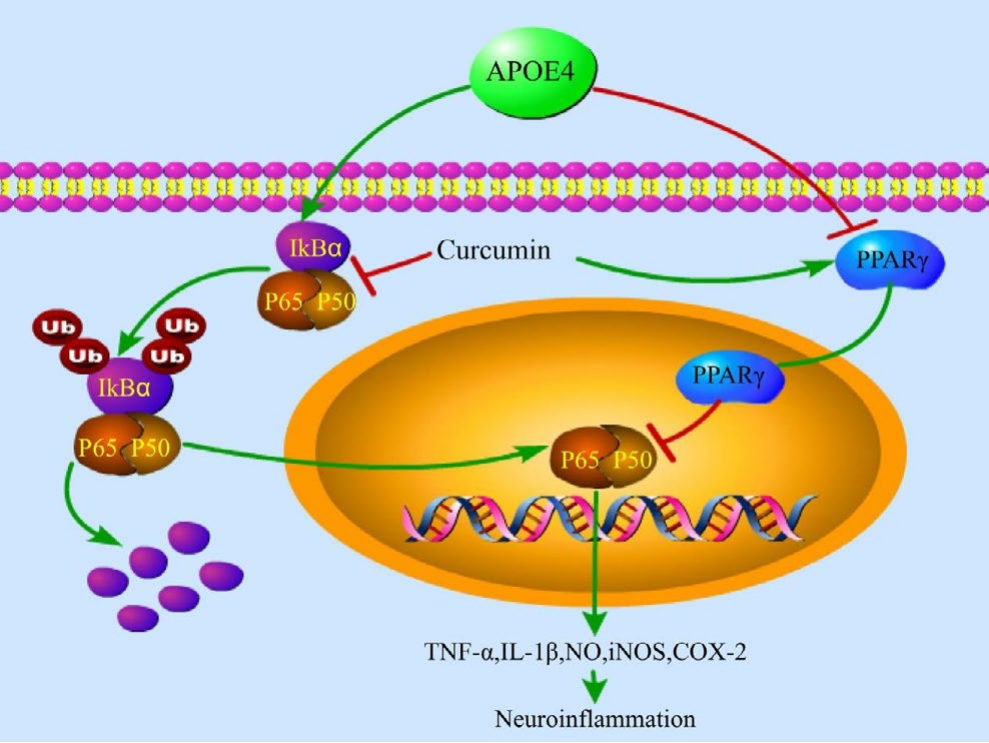
Possible mechanism of curcumin on APOE4-induced cell model

APOE4 downregulates the expression of PPARγ and activates the NF-κB signaling; curcumin prevents these processes thereby exerting neuroprotective effects.

PPARγ: Peroxisome proliferator-activated receptor-γ; APOE4: apolipoprotein E4

## Conclusion

The overexpression of APOE4 may trigger the inflammatory response by inhibiting the expression of PPARγ, thereby activating the NF-κB signaling pathway in SH-SY5Y cells. Curcumin inhibits APOE4-induced inflammation by blocking the above pathways (Figure 9). The effects of APOE4 overexpression on other glial cells and on nerve function in animals, as well as the role of curcumin and the potential mechanism still need to be further studied.
